# Inbreeding Ratio and Genetic Relationships among Strains of the Western Clawed Frog, *Xenopus tropicalis*

**DOI:** 10.1371/journal.pone.0133963

**Published:** 2015-07-29

**Authors:** Takeshi Igawa, Ai Watanabe, Atsushi Suzuki, Akihiko Kashiwagi, Keiko Kashiwagi, Anna Noble, Matt Guille, David E. Simpson, Marko E. Horb, Tamotsu Fujii, Masayuki Sumida

**Affiliations:** 1 Institute for Amphibian Biology, Graduate School of Science, Hiroshima University, Higashi-Hiroshima, Hiroshima, Japan; 2 School of Biological Sciences, Institute of Biomedical and Biomolecular Science, University of Portsmouth, Portsmouth, United Kingdom; 3 The Wellcome Trust/Cancer Research UK Gurdon Institute, The Henry Wellcome Building of Cancer and Developmental Biology, University of Cambridge, Cambridge, United Kingdom; 4 Bell Center for Regenerative Biology and Tissue Engineering and National Xenopus Resource, Marine Biological Laboratory, Woods Hole, MA, United States of America; 5 Department of Health Sciences, Faculty of Human Culture & Science, Prefectural University of Hiroshima, Hiroshima, Japan; Radboud University Nijmegen, NETHERLANDS

## Abstract

The Western clawed frog, *Xenopus tropicalis*, is a highly promising model amphibian, especially in developmental and physiological research, and as a tool for understanding disease. It was originally found in the West African rainforest belt, and was introduced to the research community in the 1990s. The major strains thus far known include the *Nigerian *and *Ivory Coast *strains. However, due to its short history as an experimental animal, the genetic relationship among the various strains has not yet been clarified, and establishment of inbred strains has not yet been achieved. Since 2003 the Institute for Amphibian Biology (IAB), Hiroshima University has maintained stocks of multiple *X*. *tropicalis *strains and conducted consecutive breeding as part of the National BioResource Project. In the present study we investigated the inbreeding ratio and genetic relationship of four inbred strains at IAB, as well as stocks from other institutions, using highly polymorphic microsatellite markers and mitochondrial haplotypes. Our results show successive reduction of heterozygosity in the genome of the IAB inbred strains. The *Ivory Coast* strains clearly differed from the *Nigerian* strains genetically, and three subgroups were identified within both the *Nigerian* and *Ivory Coast* strains. It is noteworthy that the *Ivory Coast *strains have an evolutionary divergent genetic background. Our results serve as a guide for the most effective use of *X*. *tropicalis *strains, and the long-term maintenance of multiple strains will contribute to further research efforts.

## Introduction

Amphibian species have featured prominently as experimental animals due to several key characteristics: 1) Their skeleton and internal organs are similar to those of mammals; 2) Their ovulation is controllable, resulting in many large eggs and embryos that develop robustly; 3) They display dynamic metamorphosis from tadpoles to adult frogs and 4) They have a high regenerative capacity. The African clawed frog, *Xenopus laevis*, has been the most widely used amphibian model species since the 1950s. However, due to the allotetraploid origin of its genome and its long generation time, genetic and gene function studies in *X*. *laevis* are challenging. The Western clawed frog *X*. *tropicalis*, on the other hand, is a true diploid with a shorter generation time making it better suited for genetic manipulations [1,2].

*X*. *tropicalis* was originally found in the West African rainforest belt, and is a relative of *X*. *laevis* thought to have diverged approximately 50 Million years ago [3]. Recent determination of the full nucleotide sequence of *X*. *tropicalis* offers a unique opportunity to combine forward and reverse genetics [1,4]. These advantages are accelerating the popularity of *X*. *tropicalis*. Several *X*. *tropicalis* strains have been generated and made available for use in research from various institutes and companies. However, due to its relatively short history as an experimental animal, the genetic relationships among these various strains of *X*. *tropicalis* have not been clarified, and the establishment of inbred strains has not yet been fully achieved. Inbred strains can be generated by inbreeding sibling animals over several generations, which leads to a trend toward increased homozygosity, resulting in phenotypic uniformity. Inbred strains are therefore important in reducing inter-individual variation and the number of animals used in investigations, as well as in facilitating reproducibility and uniformity of experimental results, e.g. for accurate genome sequencing, and for research on immune systems and functions [5]. Moreover, inbred strains are essential for high efficiency of advanced reverse genetic tools such as ZFN, TALEN, and CRISPR/Cas9, which have appeared in recent years [6–10]. Nonetheless, multiple strains with different genetic backgrounds are essential for genetic screens in forward genetics.

At present there are two major strains of *X*. *tropicalis*: “*Ivory Coast*” and “*Nigerian*”. According to previous reports [1,11], “*Ivory Coast*” specimens were collected from Adiopodoume, Ivory Coast by Dr. Michael Fischberg and held in the Swiss repository for *Xenopus* strains at Université de Genève until 1990, when a small group of them was brought to the University of California by Dr. Marc Kirschner. Around the same time a much larger population of the “*Nigerian*” strain was collected from Nigeria and imported directly to the Kirschner lab. Both these strains were raised under the supervision of Dr. Enrique Amaya at the University of California, and later transported to the University of Virginia, where breeding was initiated under the supervision of Dr. Robert M. Grainger [1]. A number of different *Nigerian* strains were generated in the Grainger lab and distributed to various institutions around the world, including the Institute for Amphibian Biology (IAB), Hiroshima University. At IAB, these multiple strains of *X*. *tropicalis* have been maintained and supplied to researchers as part of the National BioResource Project (NBRP) since 2003. These strains have been inbred through successive generations for more than five years. They are still vigorous in viability compared to other non-inbred strains, and are thus thought to have stabilized past the stage of inbreeding depression.

In this study we used genetic markers to investigate the genetic relationships and the magnitude of inbreeding among the strains maintained at IAB, together with stocks from the other institutes. Because some of these strains are believed to have been derived from a common founder colony and are thus closely related, we employed highly polymorphic microsatellite (i.e. Simple Sequence Length Polymorphism: SSLP) markers identified through the genome in order to construct a genetic linkage map of *X*. *tropicalis* as per [12]. We conducted genotyping of 60 SSLP markers that are spread across the whole genome and sequencing of a control region of the mitochondrial genome for the four inbred strains from IAB (*Nigerian A*, *Nigerian H*, *Golden*, and *Ivory Coast*), as well as for five *Nigerian* and three *Ivory Coast* strain stocks from the other institutes that had not been deliberately inbred. Genetic relationships and magnitude of inbreeding were estimated based on population genetic analyses.

## Materials and Methods

### Strains and stocks used in this study

**Fig 1:**
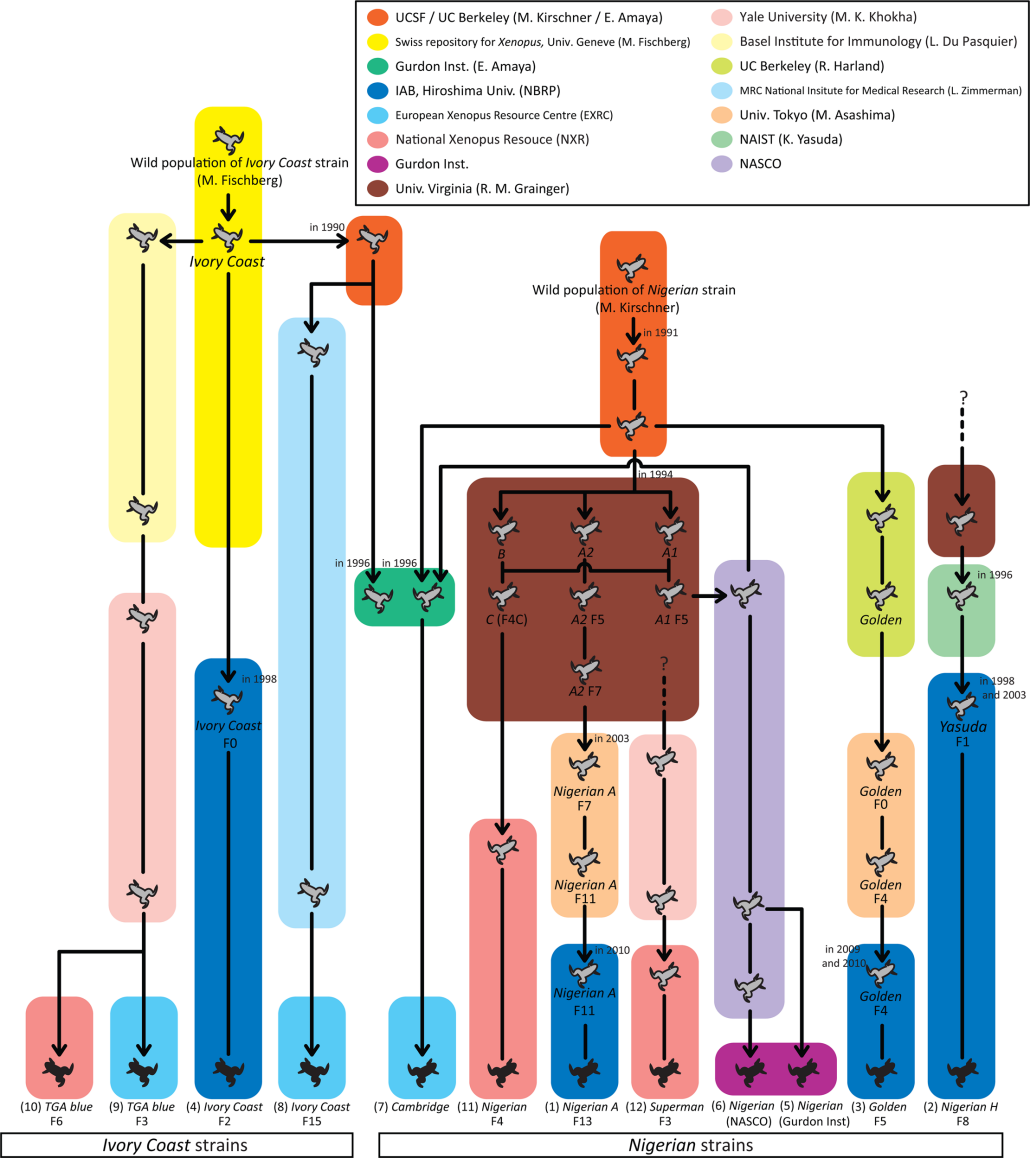
General pedigree chart of *X*. *tropicalis* strains. Frogs facing left represent the *Ivory Coast* strain and those facing right are *Nigerian* strains. Frogs colored black indicate strains used in this study. The arrows and figures next to frogs indicate transportation of animals and the years when those were held (if there is a record).

As mentioned above, the two major strains of *X*. *tropicalis* are known as *Nigerian* and *Ivory Coast*. The three *Nigerian* strains were transferred to IAB from the Grainger lab and the Harland lab by way of the University of Tokyo and the Nara Institute of Science and Technology (NAIST), while *Ivory Coast* was obtained directly from the Université de Genève. Although IAB also maintains an *Asashima* strain, it was not used in this study because it is genetically far apart and probably a different species or subspecies [13]. For comparison, we also investigated one *Nigerian* and two *Ivory Coast* stocks from the European Xenopus Resource Centre (EXRC), two *Nigerian* stocks from the The Wellcome Trust / Cancer Research UK Gurdon Institute (Gurdon Inst.) in the UK, and two *Nigerian* and one *Ivory Coast* stock from National *Xenopus* Resource (NXR) in the US. General pedigree information is summarized in Fig 1. Ten tadpoles from each colony were used for subsequent experiments and analyses, except for *Ivory Coast* from EXRC from which only two tadpoles were obtained and thus used only in the analysis based on mitochondrial genes.

#### 1) *Nigerian* (*Nigerian A*) from IAB [*NA* (IAB)]

This strain originated from a *Nigerian* strain maintained at the Grainger lab (University of Virginia, USA). Dr. Asashima (The University of Tokyo) obtained the sixth generation of the *Nigerian A2* strain used for genome sequencing by JGI as a generous gift from Dr. R. Grainger in 2003. This strain was propagated to the eleventh inbred generation in his laboratory and provided to IAB in 2014. This was simply labeled as “*Nigerian”* in a previous study [13]. For the present study we used the thirteenth inbred generation of this strain.

#### 2) *Nigerian* (*Nigerian H*) from IAB [*NH* (IAB)]

Dr. K. Yasuda (Nara Institute of Science and Technology, Japan) also obtained *X*. *tropicalis* frogs from the Grainger lab in 1996. He carried out several generations of inbreeding and provided specimens to IAB in 1998 and 2003. This was previously labeled as “*Yasuda*” [13]. We renamed this strain “*Nigerian H*” because its mitochondrial haplotype is identical to *Nigerian*. We used the eighth inbred generation of this strain.

#### 3) *Nigerian* (*Golden*) from IAB [*Golden* (IAB)]

This strain originally came from a stock maintained by Dr. E. Amaya at the Univeristy of California, Berkeley and subsequently maintained for a short period by Dr. T. Hyaes. The *Golden* strain was developed as by artificial selection for fast growth and rapid sexual maturity in the Harland lab. Dr. M. Asashima obtained this strain from Dr. R. Harland in 2005 and provided specimens to IAB in 2009 and 2010. We used the fifth inbred generation of this strain.

#### 4) *Ivory Coast* from IAB [*IC* (IAB)]

This strain was taken from a wild population in Adipodoume, Ivory Coast, and has been shown to be diploid [11]. Adult males and females were presented to Dr. K. Yoshizato (IAB at that time) in 1998 by Drs. D. Rungger and H.R. Kobel at Université de Genève.

#### 5) *Nigerian* from Gurdon Institute [*N* (Gurdon Inst.)]

This *Nigerian* strain was originally purchased from NASCO and maintained with other stocks for a while at Gurdon Inst. This strain originated from the *Nigerian* strain maintained in the Grainger lab.

#### 6) *Nigerian* from NASCO Company [*N* (NASCO)]

This *Nigerian* strain was recently purchased from NASCO by the Gurdon Inst.

#### 7) *Nigerian* (*Cambridge*) from EXRC [*Cambridge* (EXRC)]

This strain was previously maintained at the Gurdon Inst. in Cambridge by Dr. E. Amaya who kept the original *Nigerian* colony, and is now maintained at EXRC. This strain was transferred from the University of California, Berkeley to the Gurdon Inst. and maintained for sometime with the *Ivory Coast* strain and the other *Nigerian* strain purchased from NASCO (Dr. E. Amaya, personal communication).

#### 8) *Ivory Coast* from EXRC [*IC* (EXRC)]

This *Ivory Coast* strain is currently maintained at EXRC, and was a kind gift from the Lyle Zimmerman laboratory (MRC National Institute for Medical Research, Mill Hill, London), with its origin from the Marc Kirschner laboratory. We used the 15th inbred generation.

#### 9) *Ivory Coast* (*TGA blue*) from NXR [*TGA blue* (NXR)]

This *Ivory Coast* strain is currently maintained at NXR and originally derived from the F4 (C731) line of the *Ivory Coast TGA* family that was propagated at the Mustafa Khokha laboratory (Yale University). The *Ivory Coast TGA* strain originated from Dr. Louis Du Pasquier at the Basel Institute for Immunology, while the *IC* strain described above was derived from a stock at the Marc Kirschner laboratory [14]. We used the second inbred generation derived from the parental C731 line.

#### 10) *Ivory Coast* (*TGA blue*) from EXRC [*TGA blue* (EXRC)]

This Ivory Coast strain is currently maintained at EXRC, and is similar to the one maintained at NXR, both originally derived from the Khokha laboratory. We used the third inbred generation derived from the F2 lines (C411 and C412) that are parental to the F4 (C731) line.

#### 11) *Nigerian* from NXR [*N* (NXR)]

This *Nigerian* strain is currently maintained at NXR and originated from the *Nigerian C* strain (F4C, St.549) that was made by mating third generation of *Nigerian A1* and third generation of *Nigerian B* stains at the Grainger laboratory. We used the fourth inbred generation derived from the parental F4C colony.

#### 12) *Nigerian* (*Superman*) from NXR [*Superman* (NXR)]

This Nigerian strain was initially established at the Khokha laboratory by mating sublines of the *Nigerian* colony that originated from the Grainger laboratory. It was made by mating a male frog developing the nuptial pad very early with inbred *Nigerian* females. The *Nigerian Superman* strain was propagated at NXR, and is currently maintained at both NXR and EXRC. We used the third inbred generation maintained at NXR.

### Selection of SSLP markers and confirmation of physical location

In this study we focused on tetranucleotide markers due to the higher polymorphism of tetranucleotide repeats compared with other repeat types [15]. From 1,366 such markers that showed single matching with scaffolds from the JGI *X*. *tropcalis* genome assembly 4.1, we selected 66 markers (S1 Table) to achieve an even distribution interval in centimorgans (cM) in every linkage group (approx. 17 cM). Information about makers (i.e. primer sequences, repeat types, matching scaffolds, etc.) was obtained from the Genetic Map on the *X*. *tropicalis* website (http://tropmap.biology.uh.edu/). To confirm the physical location of these SSLP markers on chromosomes throughout the genome assembly, we identified their physical location on the latest JGI assembly 7.1, as well as on genes used for FISH mapping in [16]. Locations in assembly 7.1 were identified using electronic PCR (e-PCR) (Schuler 1997), with allowance for single gaps and mismatches. For the location of FISH-mapped genes, we searched Xenbase (http://www.xenbase.org/) using the gene name as the query.

### Ethics statement

The research reported herein was approved by the animal welfare and ethical review body (AWERB) of the University of Portsmouth (Licence number: PL70/7272), Hiroshima University Animal Research Committee (Approval number: G14-2), and Institutional Animal Care and Use Committees (IACUC) for NXR (Assigned protocol number: 15-02B). All tadpoles used in this study were deeply euthanized with 0.05% benzocaine in fresh water and stored individually in ethanol, and used for DNA extraction. This was carried out in accordance with the recommendations in the Guide for the Care and Use of Laboratory Animals of the Hiroshima University Animal Research Committee and under the appropriate license from the UK Home Office.

### Genotyping

Seventy tadpoles consisting of ten offspring from single matings in each of seven strains were used for DNA extraction and genotyping. DNA was extracted using NucleoSpin Tissue (Macherey & Nagel) following the standard protocol described in the instructions. Genotyping of these seventy DNA samples was carried out via M13-tail post-labelling PCR [17] using M13 primers labelled with fluorescent dyes FAM, HEX, NED, and PET (dyes for each locus and the panels are summarized in S1 Table). All forward primers of the 64 markers had an M13(-21) sequence attached on the 5’ terminal. PCRs were carried out in a 10 μl volume containing 5 μl of EmeraldAmp MAX PCR Master Mix, 0.1 μl of 1 μM forward and labeled universal primers, 0.2 μl of 2 μM reverse primer and 50 ng of genomic DNA. Thermal cycling was performed under the following conditions: 95°C for 5 min, 35 cycles each at 95°C for 30 sec, 58°C for 30 sec and 72°C for 30 sec, followed by 8 cycles each at 95°C for 15 sec 53°C for 30 sec and 72°C for 30 sec, and a final extension period of 72°C for 10 min. PCR products were electrophoresed on a 3130xl Genetic Analyzer (Life Technologies) together with GeneScan LIZ 500 (Life Technologies) as an internal size standard, and genotyped using GeneMapper 4.0 (Life Technologies).

In addition, we also amplified and sequenced approximately 2.3 kbp mitochondrial DNA fragments containing partial Cyt *b*, tRNA-Pro, tRNA-Thr, control region, and partial 12S rRNA genes for a single representative individual from each colony because the control region is the most variable in the *X*. *tropicalis* mitochondrial genome [13]. PCR for amplification was conducted in a 50 μl volume containing 25 μl of EmeraldAmp MAX PCR Master Mix (TaKaRa), 2.5 μl of 10 μM primer pair used in a previous study [13] (Trop_Cytb_CendFow and R17N1, see S2 Table) and 50 ng of genomic DNA. The amplified fragments were sequenced using five additional primers also used in [13] (see S2 Table) and the BigDye Terminator ver. 3.1 (Life Technologies) after PEG precipitation. The resultant nucleotide sequences were assembled using Phred [18] / Phrap [19] and Consed [20].

### Inference of individual kinship and relationship among the strains

Basic genotypic information of SSLP markers, including the proportion of polymorphic loci (*P*), the number of alleles (*N*_A_), the expected heterozygosity (*H*_E_) and the fixation index (*F*) was calculated using GenAlEx 6.5b3 [21]. To estimate kinship between individuals within each colony, we calculated relatedness (*r*) between every combination of ten individuals in each colony using Coancestry version 1.0.1.2 [22]. The dyadic likelihood method [23] was used for the estimator because this method outputs non-negative, realistic *r* values comparable to the relatedness estimated from physical genealogy.

To infer genetic relationships among the colonies, we calculated genetic distances based on allele frequencies of SSLP markers and substitutions of the mitochondrial genes. For SSLP markers, we calculated pairwise genetic distances and constructed phylogenetic trees based on Nei *et al*.’s *D*_A_ distance [24], and the proportion of shared alleles (*D*_ps_) [25]. Pairwise *D*_A_ and *D*_ps_ were calculated using POPTREE2 [26] and Microsatellite Analyser (MSA) 4.05 [27], respectively. Based on these distance matrixes, neighbor joining trees with 1000 bootstrap iterations were constructed using POPTREE2 for *D*A, and neighbor and consense programs of PHYLIP 3.69 [28] for *D*_PS_. For mitochondrial genes, we made two nucleotide alignment data sets to compare relationships among the studied strains and some wild populations those data were obtained from DNA database: (1) data containing approx. 2.3 kbp of the multiple genes of the twelve strains defined in this study, and (2) data containing 1.1 kbp of the control region of our twelve strains and also three wild *X*. *tropicalis* individuals each from Uyere, Nigeria (EF413678), Adipodoume, Ivory Coast (EF7413679), and Free town, Sierra Leone (EF413680). We initially aligned the nucleotide sequences using Clustal W [29] implemented in MEGA 6 [30], and eliminated any ambiguous and gap sites using Gblocks [31]. Neighbor joining trees based on p-distance with 1000 bootstrap iterations were constructed using MEGA 6.

## Results

### Physical location of markers

**Fig 2:**
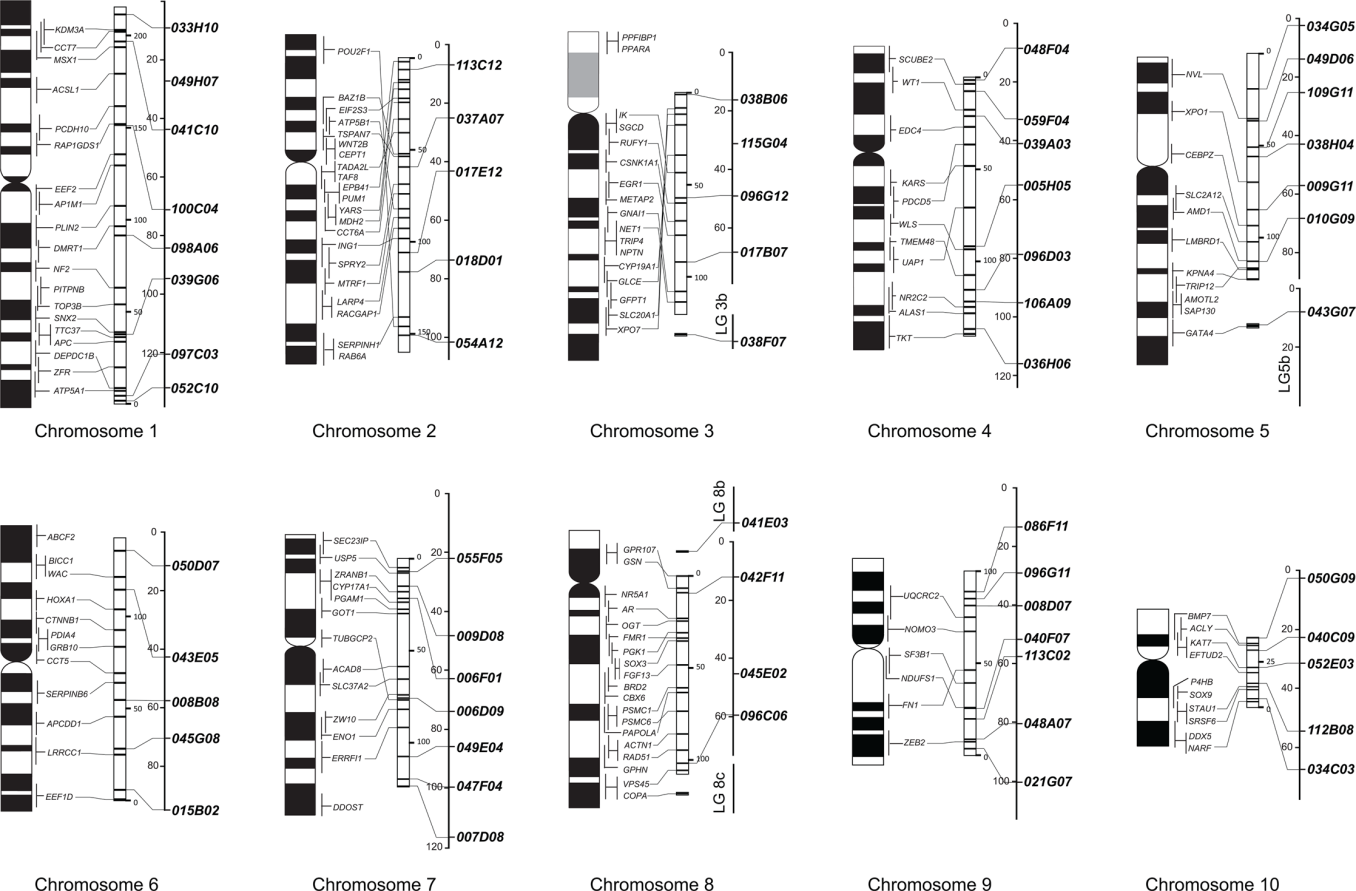
Alignment of physical and genetic linkage maps of *X*. *tropicalis*. Left = FISH cytogenetic map [16]. Middle = physical map from full nucleotide sequence data (JGI *X*. *tropicalis* genome assembly 7.1). Right = genetic map based on linkage analysis [12]. The sixty loci used in our analyses are indicated on the right. All maps are size proportional. Physical nucleotide units are mega base (Mbp), and genetic map units are centimorgan (cM).

Although the SSLP loci were found to be scattered on numerous, fragmented scaffolds in assembly 4.1, our results of ePCR showed that all loci, except for *049H07*, *115G04*, *and 045E02*, were localized on the same number of scaffolds as in assembly 7.1. *049H07 and 045E02* were not localized on any scaffold, and *045E02* of LG 3 was localized on scaffold 7. As shown in Fig 2, genome assembly based gene maps approximately corresponded to the cytogenetic gene maps made using FISH [16]. However, a contradiction between these maps was observed in parts of chromosomes 2 and 3 (Fig 2). As shown in a recent study [32], this could reflect a misassembled region of the scaffold due to unequal recombination frequencies in meiotic mapping and/or erroneous super-scaffolding based on different synteny blocks of the other species. Otherwise this might show a large chromosome inversions between the strains used for whole genome sequencing (*Nigerian* line in Grainger lab) and FISH mapping (*Yasuda* = *Nigerian H* line) [33]

### Genotyping data and individual relatedness within each colony

Of the total 66 markers genotyped in this study, we excluded six markers (*017E04*, *040B08*, *112F10*, *047C01*, *044G01*, and *021D12*) from the dataset because of ambiguity in their peaks and low-repeatability in some of the samples. In the remaining 60 markers, all loci were polymorphic [Proportion of polymorphic loci (*P*) = 1], and total number of alleles (*N*_A_) ranged from 3 (*039G06* and *034C03*) to 14 (*097C03*, *018D01*, *006D09*, *047F04*, and *086F11*), with a mean value of 9.57 over all samples. The proportion of polymorphic loci (*P*) varied among colonies, from 0.15 [*NA* (IAB)] to 0.93 [*N* (Gurdon Inst.)]. Allelic diversity (*A*) and expected heterozygosity (*H*_E_) also varied along with *P* among colonies (see Table 1). Mean fixation index (*F*) values for each colony ranged from -0.273 [*Superman* (NXR)] to 0.053 [*TGA blue* (NXR)], showing minus values in all colonies except *TGA blue* (NXR), while the mean fixation index among strains (*F*_ST_) over all loci was 0.629. Generally the three inbred *Nigerian* strains from IAB and *TGA blue* (NXR) showed smaller values for all measurements.

**Table 1:** Basic genotypic data of 60 SSLP markers in eleven *X*. *tropicalis* colonies. Abbreviations: *P*, proportion of polymofic loci; *N*_A_, number of alleles; *H*_E_, expected heterozygosity; *F*, fixation index (i.e., inbreeding coefficient).

		*NA* (IAB)				*NH* (IAB)				*Golden* (IAB)				*IC* (IAB)				*N* (Gurdon Inst)				*N* (NASCO)				*Cambridge* (EXRC)				(9) *TGA blue* (NXR)				(10) *TGA blue* (EXRC)				(11) *N* (NXR)				(12) *Superman* (NXR)				Total	
LG	No. loci	*P*	*A*	*H*_E_	*F*	*P*	*A*	*H*_E_	*F*	*P*	*A*	*H*_E_	*F*	*P*	*A*	*H*_E_	*F*	*P*	*A*	*H*_E_	*F*	*P*	*A*	*H*_E_	*F*	*P*	*A*	*H*_E_	*F*	*P*	*A*	*H*_E_	*F*	*P*	*A*	*H*_E_	*F*	*P*	*A*	*H*_E_	*F*	*P*	*A*	*H*_E_	*F*	*A*	*F*_ST_
1	8	0.25	1.25	0.122	-0.075	0.13	1.13	0.056	-0.500	0.63	1.63	0.226	-0.216	0.75	1.88	0.371	-0.120	0.88	2.00	0.403	-0.177	0.88	2.13	0.391	-0.134	0.75	2.25	0.333	-0.066	0.75	1.75	0.293	0.206	0.88	2.00	0.400	0.280	1.00	2.63	0.480	-0.024	0.63	2.38	0.320	-0.374	8.63	0.535
2	5	0.00	1.00	0.000	-	0.20	1.60	0.112	0.011	0.60	1.60	0.215	-0.173	0.60	2.00	0.280	-0.282	1.00	2.80	0.597	-0.275	1.00	3.00	0.549	-0.045	1.00	2.80	0.572	-0.191	1.00	2.00	0.418	0.253	1.00	2.00	0.406	0.123	0.80	2.20	0.332	0.384	1.00	2.20	0.430	-0.213	10.20	0.532
3	4	0.00	1.00	0.000	-	0.25	1.25	0.114	-0.538	0.25	1.25	0.094	-0.333	0.50	2.00	0.303	-0.228	1.00	3.25	0.589	-0.108	1.00	3.75	0.688	-0.140	0.75	2.75	0.481	-0.227	0.50	1.75	0.229	0.501	1.00	2.00	0.433	-0.175	1.00	3.00	0.498	-0.031	0.75	1.74	0.361	-0.409	11.25	0.522
3b	1	0.00	1.00	0.000	-	0.00	1.00	0.000	-	0.00	1.00	0.000	-	1.00	2.00	0.480	0.167	1.00	4.00	0.745	-0.342	1.00	4.00	0.725	-0.379	1.00	2.00	0.495	0.798	0.00	1.00	0.000	-	1.00	2.00	0.480	0.583	1.00	3.00	0.585	-0.538	1.00	2.00	0.408	-0.400	13.00	0.568
4	7	0.00	1.00	0.000	-	0.00	1.00	0.000	-	0.29	1.29	0.094	-0.181	0.86	2.14	0.340	-0.138	0.86	2.29	0.417	-0.151	0.71	2.00	0.282	-0.372	0.71	1.86	0.331	-0.064	0.29	1.43	0.087	-0.206	0.86	2.86	0.499	-0.088	0.86	2.43	0.383	0.037	0.67	1.67	0.215	0.004	10.00	0.651
5	6	0.33	1.33	0.140	-0.429	0.33	1.33	0.143	0.333	0.50	1.67	0.179	0.191	0.83	2.00	0.363	-0.409	0.83	2.67	0.435	-0.039	0.83	2.33	0.463	0.086	0.67	1.83	0.336	0.287	0.33	1.50	0.131	0.197	0.83	2.50	0.495	-0.090	0.67	2.00	0.328	0.143	0.50	1.67	0.239	-0.280	10.17	0.632
5b	1	0.00	1.00	0.000	-	0.00	1.00	0.000	-	0.00	1.00	0.000	-	0.00	1.00	0.000	-	1.00	2.00	0.455	0.341	1.00	2.00	0.255	-0.176	1.00	3.00	0.655	-0.527	1.00	2.00	0.480	0.167	1.00	2.00	0.420	-0.429	1.00	3.00	0.640	0.219	1.00	3.00	0.540	-0.296	8.00	0.587
6	5	0.00	1.00	0.000	-	0.00	1.00	0.000	-	0.40	1.40	0.159	-0.143	0.80	2.40	0.384	-0.059	1.00	2.60	0.492	0.020	1.00	2.40	0.517	0.040	1.00	3.60	0.620	-0.193	0.60	1.80	0.247	-0.345	0.60	1.83	0.298	-0.129	0.80	2.40	0.360	0.220	0.40	1.40	0.148	-0.101	9.80	0.619
7	7	0.42	1.57	0.206	-0.247	0.57	1.57	0.184	-0.071	0.29	1.29	0.096	-0.061	0.29	1.43	0.167	-0.500	1.00	3.29	0.624	0.040	1.00	2.71	0.510	-0.135	1.00	3.00	0.571	-0.055	0.14	1.14	0.061	-0.455	0.29	1.43	0.134	-0.025	0.57	1.57	0.223	-0.253	0.71	1.71	0.258	0.076	10.86	0.645
8	3	0.00	1.00	0.000	-	0.00	1.00	0.000	-	0.33	1.33	0.032	-0.053	0.67	2.00	0.320	-0.307	1.00	2.67	0.580	-0.021	1.00	2.67	0.521	-0.339	1.00	3.00	0.555	0.049	0.33	1.33	0.125	0.200	1.00	2.67	0.573	-0.631	0.33	1.33	0.140	0.048	0.66	2.00	0.318	-0.260	9.00	0.513
8b	1	0.00	1.00	0.000	-	0.00	1.00	0.000	-	0.00	1.00	0.000	-	0.00	1.00	0.000	-	1.00	3.00	0.645	-0.240	1.00	3.00	0.620	-0.613	0.00	1.00	0.000	-	0.00	1.00	0.000	-	0.00	1.00	0.000	-	1.00	3.00	0.505	-0.188	1.00	3.00	0.500	-0.333	6.00	0.701
9	7	0.14	1.14	0.069	-0.250	0.57	1.71	0.229	-0.368	0.71	1.43	0.214	-0.805	0.71	1.86	0.361	-0.378	0.86	2.57	0.458	-0.277	0.86	2.71	0.514	-0.449	0.86	2.43	0.427	-0.248	0.29	1.29	0.114	-0.381	0.71	2.00	0.339	-0.341	0.86	2.00	0.348	-0.353	1.00	2.00	0.419	-0.394	8.86	0.455
10	5	0.20	1.20	0.084	-0.429	0.00	1.00	0.000	-	0.20	1.20	0.084	-0.429	0.60	1.80	0.237	0.182	1.00	2.80	0.529	-0.232	1.00	2.40	0.471	-0.171	1.00	2.40	0.492	-0.518	0.40	1.40	0.171	-0.083	0.80	2.00	0.410	-0.227	0.20	1.20	0.019	-0.053	0.60	1.60	0.299	-0.633	7.40	0.555
Total	60	0.15	1.17	0.069	-0.270	0.22	1.27	0.087	-0.163	0.38	1.40	0.141	-0.219	0.65	1.90	0.306	-0.203	0.93	2.67	0.509	-0.117	0.92	2.57	0.477	-0.175	0.85	2.50	0.446	-0.122	0.45	1.52	0.182	0.053	0.77	2.10	0.385	-0.078	0.75	2.15	0.337	-0.170	0.71	1.90	0.308	-0.273	9.57	0.629

**Fig 3:**
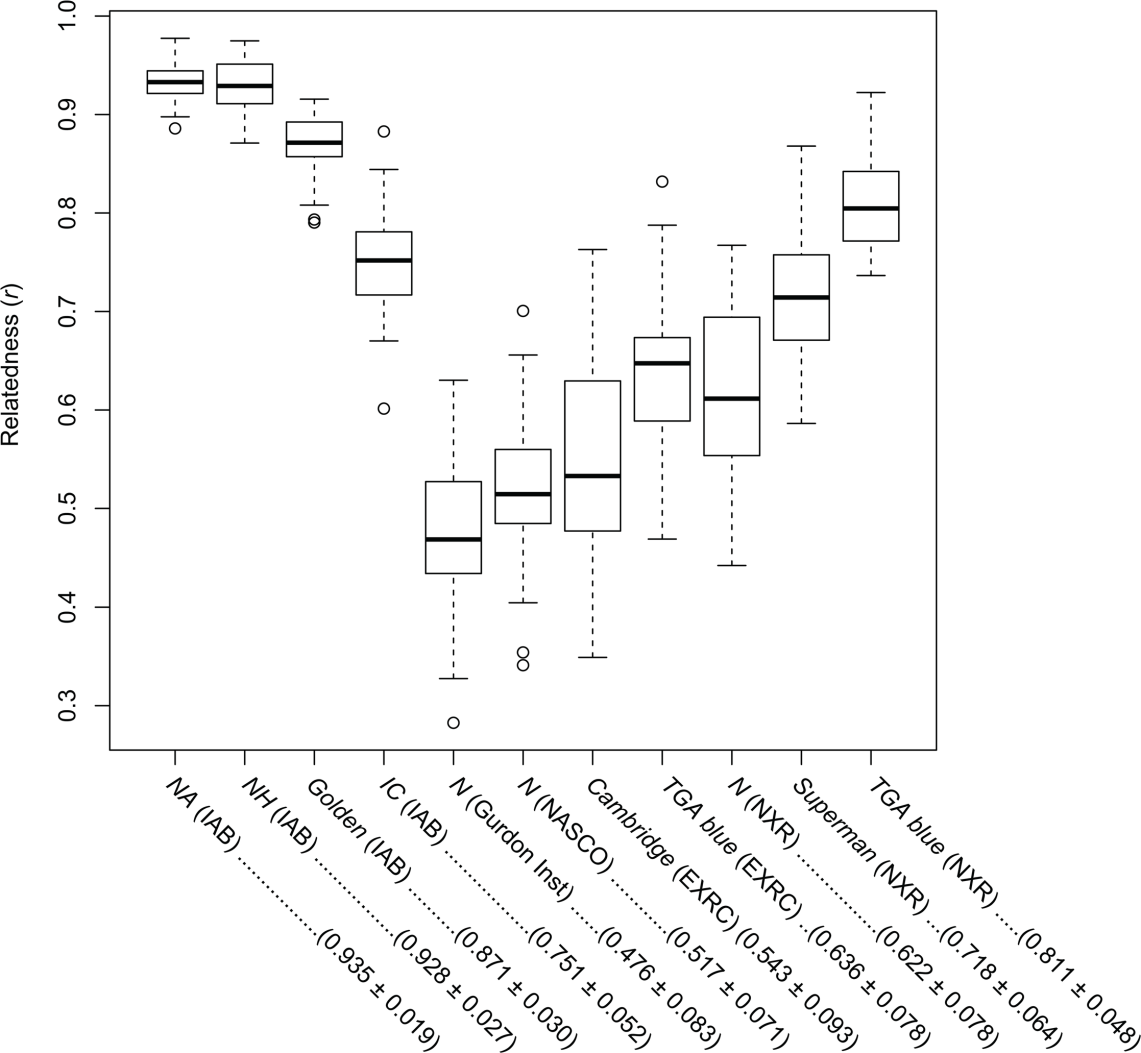
Observed distribution of pairwise *r* estimates among *X*. *tropicalis* strains. Box plots represent the distribution of relatedness values (*r*) of each strain. Mean and SD values are described in the parentheses below the labels.

Based on these genotype data, we estimated pairwise genetic relatedness (*r*) for every combination of 10 individuals in each colony. In the same manner as above, the three inbred *Nigerian* strains from IAB and *TGA blue* (NXR) showed higher mean *r* values ranging from 0.811 [*TGA blue* (NXR)] to 0.935 [*NA* (IAB)], while some of the other non-inbred colonies, *N* (Gurdon Inst.), *N* (NASCO), and *Cambridge* (EXRC) showed relatively lower mean *r* values ranging from 0.476 [*N* (Gurdon Inst.)] to 0.543 [*Cambridge* (EXRC)] (Fig 3).

### Genetic relationships among colonies

Genetic distance matrixes *D*_A_ and *D*_ps_, based on the genotypic data of SSLP, both showed large values between the major strain groups, *Nigerian* and *Ivory Coast* (means of 0.934 and 0.941 for *D*_A_ and *D*_ps_), with moderate values among the eight *Nigerian* strains (means of 0.635 and 0.836 for *D*_A_ and *D*_ps_) and the three *Ivory Coast* strains (means of 0.633 and 0.698 for *D*_A_ and *D*_ps_) (Table 2). For mitochondrial DNA, we defined sequences of mitochondrial genes including partial Cyt *b*, tRNA-Pro, tRNA-Thr, control region, and partial 12S rRNA for all twelve strains [including *IC* (EXRC) which was not used for analyses based on SSLP] and deposited these in the DDBJ database with the accession number LC060860 –LC060871. As shown in Fig 3B, we found tandem repeats consisting of a 92 bp motif sequence at the 5’ end of the control region, together with the other conserved region, origin of heavy strand replication (O_H_) and conserved sequence blocks (CSB-1, -2, and -3) by comparison with those in *X*. *laevis* (accession number: M10217). The haplotypes of *Nigerian* strains were completely identical with the previously defined mitochondrial genome of *X*. *tropicalis* (AY789013) (2,331 nucleotide sites after removing primer sites), while the haplotypes of the three *Ivory Coast* strains were different from the *Nigerian* strains and varied among the four strains investigated here except for an identical haplotype of the two *TGA blue* strains. The haplotype of *IC* (IAB) in particular was longer in nucleotide length (2,522 nucleotide sites after removing primer sites) due to two additional duplicated motifs in the tandem repeat region.

**Table 2:** Pairwise *D*_A_ (below the diagonal) and *D*_ps_ (above the diagonal) distances among *X*. *tropicalis* colonies.

	(1)	(2)	(3)	(4)	(5)	(6)	(7)	(8)	(9)	(10)	(11)
(1) *NA* (IAB)	-	0.721	0.797	0.965	0.712	0.698	0.535	0.978	0.963	0.708	0.704
(2) *NH* (IAB)	0.745	-	0.859	0.915	0.780	0.801	0.761	0.933	0.934	0.795	0.810
(3) *Golden* (IAB)	0.829	0.836	-	0.948	0.698	0.659	0.752	0.922	0.934	0.791	0.744
(4) *IC* (IAB)	0.971	0.911	0.948	-	0.971	0.967	0.964	0.805	0.818	0.950	0.970
(5) *N* (Gurdon Inst)	0.790	0.690	0.585	0.957	-	0.271	0.665	0.938	0.946	0.717	0.650
(6) *N* (NASCO)	0.783	0.714	0.551	0.955	0.137	-	0.641	0.910	0.946	0.692	0.668
(7) *Cambridge* (EXRC)	0.650	0.678	0.683	0.951	0.569	0.541	-	0.916	0.930	0.624	0.561
(8) *TGA blue*(NXR)	0.968	0.926	0.899	0.756	0.925	0.887	0.891	-	0.470	0.909	0.920
(9) *TGA blue* (EXRC)	0.955	0.930	0.923	0.793	0.924	0.919	0.905	0.350	-	0.927	0.918
(10) *N* (NXR)	0.628	0.703	0.726	0.939	0.620	0.601	0.519	0.932	0.948	-	0.526
(11) *Superman* (NXR)	0.626	0.755	0.670	0.959	0.541	0.568	0.433	0.938	0.944	0.615	-

**Fig 4:**
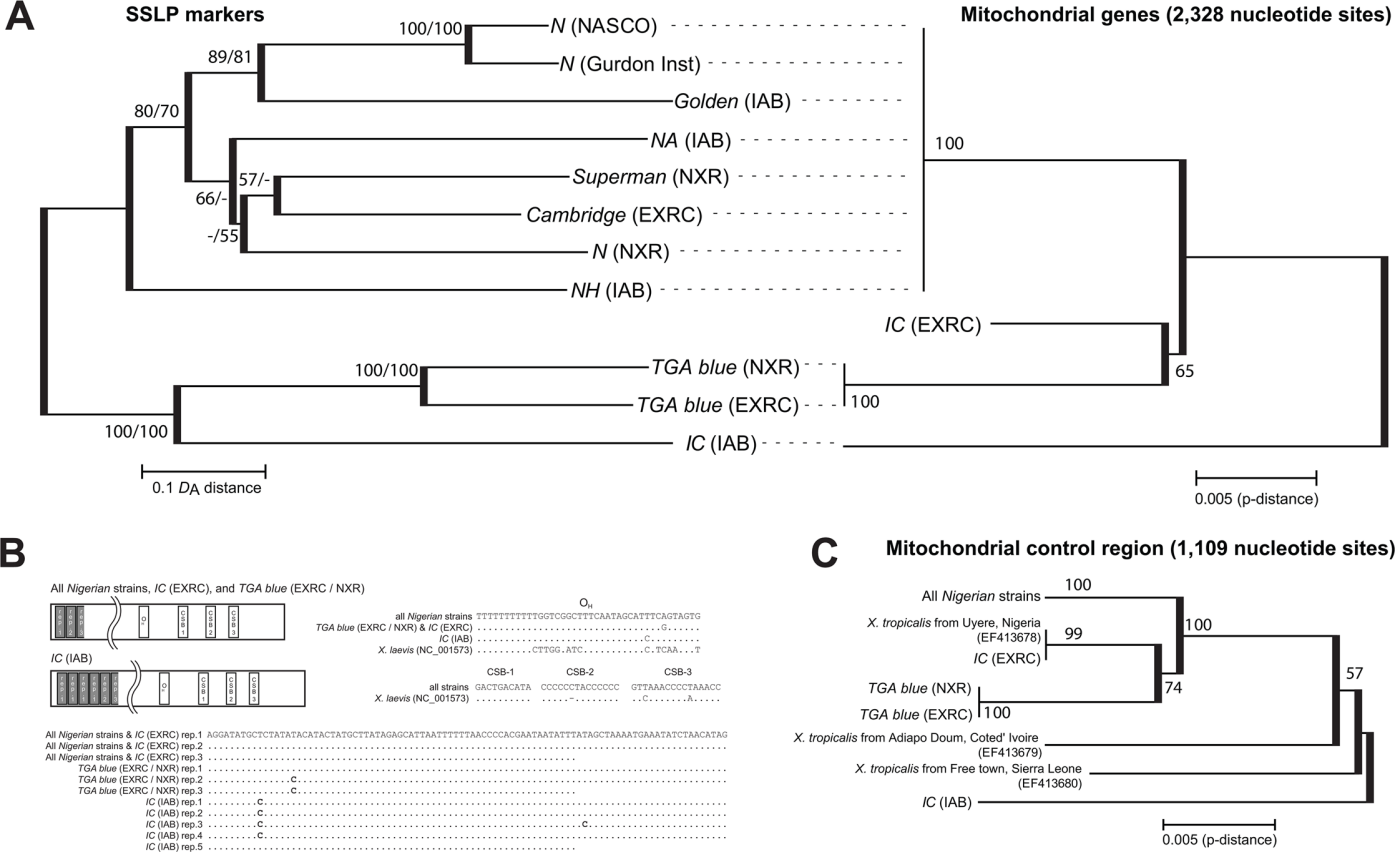
Neighbor joining trees and comparison of the control region structures of mitochondrial genomes. (A) Neighbor joining trees of the strains based on genetic distances (*D*_A_) of SSLP data (left) and p-distance of mitochondrial haplotypes (2,328 nucleotide sites) (right). Numbers on branches indicate percent bootstrap probability (>50%). The values for SSLP tree are given in order for *D*_A_/*D*_PS_. (B) Comparison of the control region structures of mitochondrial genomes. Structures of the control region and alignments of identified putative origin of H-strand replication (O_H_), conserved sequence blocks (CSB-1-3), and repeat motifs are shown. (C) Neighbor joining tree of the strains and wild individuals based on p-distance of mitochondrial haplotypes (1,109 nucleotide sites).

These divergent patterns in the genotypic data of SSLP and the haplotypes of mitochondrial DNA were basically reflected in the phylogenetic trees based on these data. For the phylogenetic trees based on genetic distance matrixes of SSLP genotypic data, both *D*_A_ and *D*_ps_ showed similar results with higher bootstrap values, thus we show only the tree based on *D*_A,_ with bootstrap values in both *D*_A_ and *D*_ps_ (Fig 4A). In this tree, the two major strain groups were firstly diverged with high bootstrap values (100% in both *D*_A_ and *D*_ps_). Within the *Nigerian* group, *NH* (IAB) was the first to diverge, followed by a split into two groups: one consisting of *Golden* (IAB), *N* (Gurdon Inst.) and *N* (*NASCO*), and the other consisting of *NA* (IAB), *N* (NXR), *Cambridge* (EXRC) and *Superman* (NXR). Although this grouping was clearly supported with relatively higher bootstrap values (> 70%), the relationships within the latter group were ambiguous with mostly lower bootstrap values around 50% or less. *N* (Gurdon Inst.) and *N* (NASCO) in particular were genetically almost identical, showing the smallest values of 0.137 and 0.271 for *D*_A_ and *D*_ps_, respectively (Table 2). Within the *Ivory Coast* group, *IC* (IAB) was clearly diverged from the two *TGA blue* strains with high bootstrap values (100% in both *D*_A_ and *D*_ps_). The phylogenetic tree of mitochondrial DNA haplotypes of all twelve strains was constructed based on 2,328 nucleotide sites after elimination of ambiguous and gap sites (Fig 4B). Although this tree is somewhat similar to SSLP tree, the *IC* (IAB) was the first to diverge when the midpoint of the tree length was rooted, reflecting the large difference in the nucleotide sequence of its haplotype. However the bootstrap support of a clade consisting of *IC* (EXRC) and *TGA blue* haplotypes was not so high (= 65%), and thus the evolutionary relationships of these haplotypes are still unclear. We also conducted analyses adding three haplotypes from three wild populations deposited in the DNA database. Another tree was constructed based on 1,109 nucleotide sites after the elimination of missing sequence and ambiguous sites in these additional data. In this tree, the relationships among strains were basically the same as the above tree. Interestingly, the haplotype of the individual from Uyere, Nigeria was found to be identical to that of *IC* (EXRC), while the haplotypes of the individuals from Adiapodoum, Ivory Coast and Free town, Sierra Leone were not grouped with any others and shown to be diverged at an intermediate position between *IC* (IAB) and the other *Ivory Coast* strains.

## Discussion

### Inbreeding ratios of the strains

**Fig 5:**
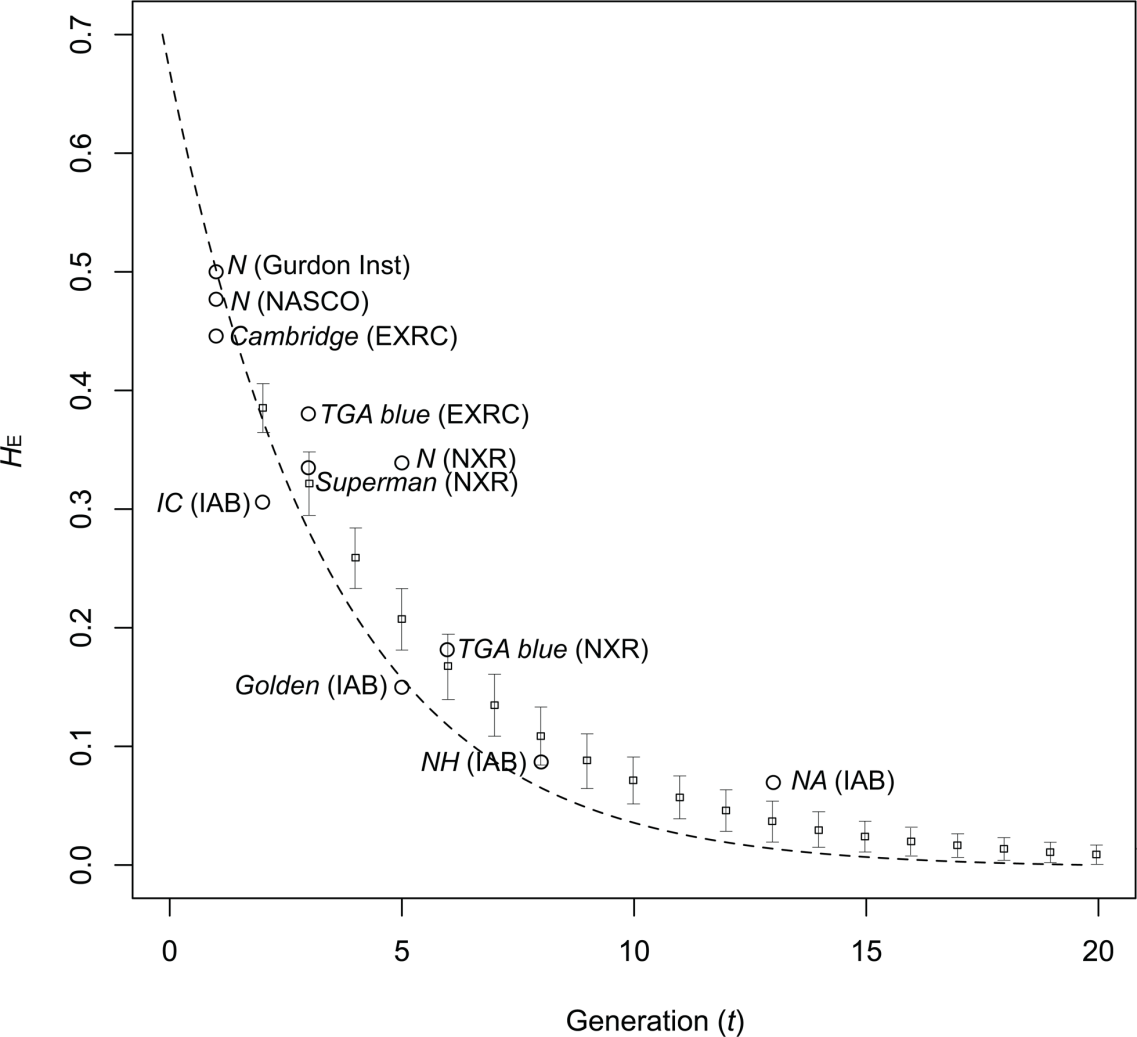
Plot of expected heterozygosity (*H*_E_) against generation number (*t*). The curved dotted strain indicates theoretical reduction of heterozygosity via single brother-sister matings for every generation, calculated from the following equation: $H_{\text{E}}=H_{0}{\left(1-\frac{1}{{2N}_{\text{e}}}\right)}^{t}$, where *H*_0_ is the heterozygosity of the founder colony, and *N*e is effective population size (i.e., number of individuals that contribute to breeding). *H*_E_ of F0 of *N* (Gurdon Inst.) (0.509) and 2 are used for *H*_0_ and *N*e, respectively. Open rectangles indicate mean *H*_E_ estimated from individual based simulation using VORTEX version 10 [43]. During simulation, allele frequencies of *N* (Gurdon Inst.) were used as initial allele frequency, and single brother-sister mating was conducted in every generation. The simulation was repeated one hundred times. Bar indicates standard deviation.

In this study we estimated genetic data on SSLP markers from all chromosomes. Because each chromosome is operated independently during meiosis, it is considered that the lineages of the chromosomes sometimes differ, especially for recent crossing between different strains. Therefore, to check the heterogeneities of these measurements across the chromosome, we conducted exact tests both in each colony and by combining all samples, but no significant differences were detected (data not shown). Comparison of the measurements among colonies highlighted lower polymorphism values for the three inbred *Nigerian* strains, *NA* (IAB), *NH* (IAB), and *Golden* (IAB). *NA* and *NH* in particular showed the lowest and the second lowest *H*E values, reflecting their longer history of brother-sister mating. Within *Ivory Coast* strain, *TGA blue* (NXR) and *IC* (IAB) showed equally lower values. Inbreeding coefficients or *F* values are estimated by dividing residual observed heterozygosity (*H*_O_) by *H*_E,_ and thus become minus when *H*_O_ exceeds *H*_E_. All samples used in this study were offspring of single brother-sister pairs, and the number of alleles was limited. In this situation, allelic frequency fluctuates due to sampling, and *F* for most loci of *NA* (IAB) and *NH* (IAB) could not be estimated due to a lack of multiple alleles. Even taking into account these shortcomings, however, observation of heterozygote excess and minus *F* values in most cases might be explained by heterozygote advantage on marker linked loci. In either case, *F* was not an appropriate estimator for inferring the inbreeding ratio in this study. Thus we estimated pairwise relatedness (*r*) within each colony. In theory, *r* values are estimated from real pedigrees and become 0.5 when the relationship is parent-offspring or brother-sister, and 1.0 when the individuals are identical clones. Our estimated *r* values within each colony showed a mean of more than 0.5 (Fig 3), which is consistent with the physical sister-brother relationship of the samples. Of these, the three *Nigerian* strains maintained at IAB showed significantly higher *r* values, reflecting a smaller value of *H*_E_ (Table 1). In particular, individuals in the *NA* and *NH* strains are nearly identical clones, showing *r* values of almost 1.0 (Fig 3). These results suggest that these four IAB strains have been successfully maintained to achieve inbred strains. *H*_E_ values for these strains, however, deviated somewhat from the theoretical and simulation-based predictive reduction curve when brother-sister matings are conducted in every generation (Fig 5). Because loss of alleles and reduction of heterozygosity are stochastic processes, such deviation can be caused by chance. In addition, different allele frequency and heterozygosity in the ancestral colony or at some specific loci might have caused this discrepancy between strains. In order to reduce heterozygosity in *NA* and *NH* to establish inbred strains on the same level with the mouse, which retain heterozygosity less than 0.02, at least five or six more generations will be necessary. Alternatively, a more rapid establishment of inbred strains may be accomplished using the gynogenetic technique [34,35].

### Genetic relationships among colonies

Our phylogenetic tree based on the mitochondrial haplotype and genotypic data on SSLP markers showed clear divergence of the *Nigerian* strains from the *Ivory* Coast strains, in agreement with a previous report which stated that *NA* (IAB) and *NH* (IAB) separated from *IC* (IAB) about 1.8 million years ago [13]. However, *D*_ps_ values based on SSLP makers between *Nigerian* and *Ivory Coast* strains are not equal to 1.0, and thus there were a few shared alleles between them. Although the existence of the shared alleles is also mentioned in the other study based on SNPs in 17 genes involved in development processes [36], the proportion of shared alleles were lower in our SSLP data, ranging from 9% to 2% with a mean of 6%. Because there are no documents refer to crossing between them, these shared alleles would be derived from the shared ancestral polymorphisms retained in the wild populations [37,38], and the higher proportion of that in SNPs should be over estimation due to recurrent mutations (independent occurrence of the mutation causing same nucleotide) between independent lineages or the balancing selection within lineages [39]. In addition, observation of a single mitochondrial haplotype shared by all *Nigerian* strains used in this study, suggests that they are descendants of a single founding colony, or else originated from very closely related colonies. On the contrary, multiple divergent mitochondrial haplotypes were found among the *Ivory Coast* strains, even though they all originally came from the same institute, the Swiss repository for *Xenopus*, Université de Genève under the direction of Dr. M. Fischberg. These haplotype divergences are likely to reflect intraspecific variations accumulated by long-term isolation of geographically isolated populations. Although we could not trace back precisely from which original wild populations founding individuals were taken due to the lack of records, our phylogenetic tree including database data suggests that the origin of the four *Ivory Coast* strains must be three different wild populations at least, and the mitochondrial haplotype of *IC* (EXRC) in particular is likely to have originated from a wild population near Uyere, Nigeria which has the same haplotype (Fig 4C). The haplotype of *IC* (IAB) is the most diverged one, this is corroborated by the increased number of repeat motifs in the control region, and thus it is most likely to have originated from another local population. A previous study [3], supported a different genetic relationship between Ivory Coast and Sierra Leone (Sierra Leone has a rather closer relationship with Nigerian than Ivory Coast), thus the relationship is still unclear. Alternatively, there might be genetically different populations residing within the same country or region. In either case, these findings from the mitochondrial data indicate that the original stocks maintained at Geneva included genetically heterogeneous colonies obtained from different localities in Western Africa. It should be noted however, that these data do not mean that each *Ivory Coast* strain originated purely from their respective wild population without crossing with any other strains. Actually SSLP marker data showed a closer relationship among three *Ivory Coast* strains, *IC* (IAB), *TGA blue* (NXR), and *TGA blue* (EXRC) with lower genetic distance values together with higher ratios of shared alleles among them, which rather suggest that they might have been partly mixed during earlier generations while still at Geneva.

Within *Nigerian strains*, SSLP data showed that *NH* (IAB) was the first to diverge from the other *Nigerian* strains and can be considered to be the most genetically remote within the *Nigerians*. Although the exact origin of the *NH* (IAB) strain is unknown, this relationship may be reflected in the fact that the founder individuals of *NH* (IAB) were transferred to NAIST from the Grainger lab quite soon after acquisition of *Nigerian* animals and before they were bred. Within the remaining *Nigerians*, two distinct groups were confirmed, represented by two strains: *NA* (IAB) and *Golden* (IAB). The *NA* (IAB) was grouped with *N* (NXR), *Superman* (NXR), and *Cambridge* (EXRC) which is derived from the colony previously maintained by Dr. E. Amaya. The original strain of these except *Cambridge* (EXRC) was in the Grainger lab, and also introduced by Dr. E. Amaya. *Cambridge* (EXRC) was previously maintained at the Gurdon Institute together with another *Nigerian* strain, which was bought from NASCO and also originated in the Grainger lab (Fig 1). Considering this background history, they were most likely derived from a common ancestral colony maintained at the Grainger lab after separation of the ancestor of the *Golden* (IAB) strain. However, there is uncertainty on the finer genetic relationships of strains within this group. This may be because the ancestral colonies of these strains were separated simultaneously from a single colony, and/or they had been crossed each other in their earlier generations. Whereas *Golden* (IAB) was grouped with *N* (Gurdon Inst.) and *N* (NASCO) and those two strains are almost genetically identical showing little genetic distance and sharing most alleles. This is reasonable because both were obtained from NASCO in a short time interval and are likely to be derived from the same stock. The exact origin of the stock in NASCO is not clear, but it also is likely to have been derived from the ancestral colony of the *Golden* strain, the relationship between the two being close. According to the chart available on the Harland lab website, the *A1* strain was transported to NASCO (Fig 1) and thus might be their common ancestor.

On the whole, our tree showed substantial genetic distances even within the same *Nigerian* and *Ivory Coast* strain groups. This can be is explained by the fixation of different alleles through genetic drift after translocation and isolation of those colonies in the same as seen in natural populations. This fixation of different alleles through genetic drift or selection among different colonies will contribute to generation of more suitable strains for screening gene function and elucidating genetic systems. The variety of fixed alleles generates the variations in phenotypic character among the different strains, and this is useful for comparative analysis in identifying gene function and genetic regulatory systems. In parallel with such diversification of strains, the strains with a shorter generation times such as *Golden* and *Superman* will make genetics more convenient in this model animal. In addition, because all *X*. *tropicalis* strains clearly originate from only two separate wild colonies, the genetic variations retained in each strain are tractable and understandable without having to take into account a complex genetic background derived from hybridization or the evolutionary context in the wild. This is significantly different from the other vertebrate experimental animals such as the mouse and zebrafish, which originated from hybridized domesticated colonies [40–42]. Therefore maintenance of local colonies of *X*. *tropicalis* at each institute should be continued over the long-term, as those will be invaluable as resource for further research.

## Conclusion

Our analyses, based mainly on SSLP markers, uncovered the genetic character and relationship among viable *X*. *tropicalis* strains maintained in various countries. The inbred strains maintained at IAB, Hiroshima University show much lower heterozygosities than strains maintained elsewhere. The genetic relationship between the *Nigerian* strains and *Ivory Coast* strains is apparently distinct, and three subgroups were confirmed within both *Nigerian* and *Ivory Coast* strains. The *Ivory Coast* strains in particular have evolutionally divergent genetic backgrounds. Finally, we should stress that the information presented here will become an important guideline for use of these strains in various laboratory experiments, especially for identification of gene function in developmental and physiological studies. In particular the inbred lines will be critical for generating lines with site-specific insertions based around genome editing techniques, in *X*. *laevis* this requires the inbred J-strain to work efficiently (N. Gadjasik, C. Sharpe and M. Guille, manuscript in preparation).

## Supporting Information

S1 Table Information of SSR loci used in this study. Original data were obtained from the website Genetic Map of *X*. *tropicalis* (http://tropmap.biology.uh.edu/).
(XLSX)

S2 Table Primers used for amplification and sequencing mitochondrial DNA. Seven primers used in this study and also in [13].
(XLSX)
